# Viral FLIP blocks Caspase-8 driven apoptosis in the gut *in vivo*

**DOI:** 10.1371/journal.pone.0228441

**Published:** 2020-01-30

**Authors:** Barbara Ruder, Claudia Günther, Michael Stürzl, Markus Friedrich Neurath, Ethel Cesarman, Gianna Ballon, Christoph Becker

**Affiliations:** 1 Department of Medicine 1, University of Erlangen-Nürnberg, Erlangen, Germany; 2 Division of Molecular and Experimental Surgery, Department of Surgery, University of Erlangen-Nürnberg, Erlangen, Germany; 3 Department of Pathology and Laboratory Medicine, Weill Medical College of Cornell University, New York, NY, United States of America; 4 Department of Pathology and Laboratory Services, Cooper University Health Care, Camden, NY, United States of America; Toho University Graduate School of Medicine, JAPAN

## Abstract

A strict cell death control in the intestinal epithelium is indispensable to maintain barrier integrity and homeostasis. In order to achieve a balance between cell proliferation and cell death, a tight regulation of Caspase-8, which is a key player in controlling apoptosis, is required. Caspase-8 activity is regulated by cellular FLIP proteins. These proteins are expressed in different isoforms (cFLIP_long_ and cFLIP_short_) which determine cell death and survival. Interestingly, several viruses encode FLIP proteins, homologous to cFLIP_short_, which are described to regulate Caspase-8 and the host cell death machinery. In the current study a mouse model was generated to show the impact of viral FLIP (vFLIP) from Kaposi’s Sarcoma-associated Herpesvirus (KSHV)/ Human Herpesvirus-8 (HHV-8) on cell death regulation in the gut. Our results demonstrate that expression of *vFlip* in intestinal epithelial cells suppressed *cFlip* expression, but protected mice from lethality, tissue damage and excessive apoptotic cell death induced by genetic *cFlip* deletion. Finally, our model shows that *vFlip* expression decreases *cFlip* mediated Caspase-8 activation in intestinal epithelial cells. In conclusion, our data suggests that viral FLIP neutralizes and compensates for cellular FLIP, efficiently counteracting host cell death induction and facilitating further propagation in the host organism.

## Introduction

A strict regulation of cell death and proliferation is necessary to maintain tissue homeostasis in the gut. On the one hand, stem cells at the crypt base continuously proliferate, which provides the basis for the enormous self-renewing capacity of the intestinal epithelium. On the other hand, fully differentiated cells are shed into the intestinal lumen at the villus tip [1, 2]. The process of cell shedding is mediated by highly regulated mechanisms. These include the regulation of tight junction proteins to seal the gap in the epithelial barrier and the induction of detachment-dependent apoptosis of the shed cell [3]. One of the central molecules that regulates cell death in the intestinal epithelium is Caspase-8, a cysteine protease which activates a downstream signaling cascade, culminating in apoptosis, a type of non-inflammatory programmed cell death [4]. Interestingly, pharmacologic inhibition or genetic deletion of Caspase-8 in intestinal epithelial cells (IECs) not only blocked apoptosis, but was shown to induce another type of necrotic, inflammatory, programmed cell death which was identified as RIPK3-dependent necroptosis [5, 6]. Caspase-8 can be activated by death-receptor signaling at the cellular surface. Activation of this signaling cascade mediates formation of Caspase-8 homodimers and a two-step autocatalytic cleavage, resulting in full maturation of the enzyme. Active Caspase-8 can then finally trigger the downstream apoptosis cascade [4]. Caspase-8 activation is tightly controlled by cellular FLIP proteins, which are mainly expressed in two different isoforms in humans, cFLIP_long_ and cFLIP_short_ [7, 8]. cFLIP proteins share structural homologies with Caspase-8, as cFLIP and Caspase-8 both are characterized by two N-terminal DED domains. cFLIP_long_ moreover comprises an inactive pseudocaspase-domain, sharing high homology with the catalytic domain of Caspase-8 [7]. Due to lack of a functional caspase-domain, binding of cFLIP_long_ to Caspase-8 only induces a first cleavage step, resulting in partial Caspase-8 activation. Partial activation does not enable Caspase-8 to initiate the downstream apoptosis cascade, however it allows cleavage and therefore inactivation of the necroptosis mediator RIPK3 [9, 10]. Binding of cFLIP_long_ to Caspase-8 therefore mediates cell survival by blocking both apoptosis and necroptosis. On the contrary, binding of cFLIP_short_ to Caspase-8 completely blocks Caspase-8 maturation and activation [9]. Blocking of Caspase-8 by cFLIP_short_ was shown to mediate cell survival due to inhibition of apoptosis. However, instead of apoptosis, there is the potential for RIPK3-mediated necroptosis to be induced in several cell types [9, 11, 12]. Interestingly there are several herpes- and poxviruses that express viral FLIP proteins, which share structural homologies to cFLIP_short_. These proteins are able to block apoptosis by interfering with the host cell death machinery [13]. Bélanger *et al*. showed that the Kaposi’s Sarcoma-associated Herpesvirus (KSHV)-/Human Herpesvirus 8 (HHV8)-vFLIP is able to bind to Caspase-8 to block its maturation and activation in a cell line *in vitro*. Therefore vFLIP was considered a viral Caspase-8 inhibitor [14]. It is tempting to speculate that during evolution, viruses, like HHV8 gained the ability to express these viral homologues in order to actively regulate the host cell death machinery and to facilitate propagation and further spreading. In addition to blocking apoptosis, inhibition of Caspase-8 by vFLIP might also induce RIPK3-dependent necroptosis in IECs [15]. Interestingly, mice constitutively expressing *vFlip* in IECs (*vFlip*^*IEC-tg*^ mice) showed a phenotype comparable to *Casp8*^*ΔIEC*^ mice. These mice are characterized by intestinal inflammation, Paneth cell loss and increased cell death, suggesting that vFLIP, similar to cFLIP_short_, inhibits Caspase-8 maturation and activation. However, in contrast to *Casp8*^*ΔIEC*^ mice, cell death in *vFlip*^*IEC-tg*^ mice was not dependent on RIPK3, suggesting that IECs did not die due to classical RIPK3-mediated necroptosis [5, 16]. The aim of the present study was to investigate if viral FLIP can compensate for cFLIP in IECs by undertaking its Caspase-8-regulating functions. With this goal in mind, we took advantage of a short term apoptosis model, characterized by massive apoptotic cell death in IECs due to inducible deletion of *cFlip* [6]. In this model, we could demonstrate that expression of viral *Flip* in IECs protected mice from intestinal epithelial cell death and lethality induced by *cFlip* deletion. This was mediated by reduced levels of Caspase-8-mediated apoptosis and barrier destruction, suggesting that HHV8-vFLIP compensates for cFLIP regarding cell death regulation in the gut during infection.

## Material and methods

### Mice

The generation of Rosa26.vFLIP, *vFlip*^*IEC-tg*^, *cFlip*^*iΔIEC*^ and VillinCreERT2 mice was described earlier [6, 16–18]. To generate *vFlip*^*iIEC-tg*^ mice with an inducible *vFlip* expression in IECs, Rosa26.vFLIP mice were crossed to VillinCreERT2 mice. To generate mice with an inducible deletion of *cFlip* and a transgenic expression of *vFlip* in IECs, *cFlip*^*iΔIEC*^ mice were crossed with *vFlip*^*iIEC-tg*^ mice. To induce deletion of floxed alleles or the stop cassette in mice which express an inducible CreERT2 recombinase, mice were injected intraperitoneally with 0.5 mg tamoxifen prediluted in Ethanol and dissolved in 100μl sunflower oil for two to three consecutive days. In survival experiments, mice were euthanized for ethical reasons if body weight loss exceeded 20% during the indicated experimental time period. Mice were routinely screened for pathogens and experiments were performed by skilled experimenters trained according to FELASA guidelines. The health and behavior of the animals was monitored daily. Animal protocols were approved by the Institutional Animal Care and Use Committee of the Regierung von Unterfranken.

### Histology and immunohistochemistry

Histopathological examinations were performed after Mayer’s H&E staining. Immunofluorescence staining was performed on formalin-fixed paraffin-embedded tissue by using the Tyramide Signal Amplification (TSA) Cy3 system (Perkin&Elmer) according to the manufacturer’s protocol. The following primary antibodies were used: Anti-Villin (Santa Cruz), anti-β-Catenin (Cell Signaling), anti-Caspase-8 (Cell Signaling) and anti-Cleaved Caspase-8 (Cell Signaling). A biotinylated anti-rabbit secondary antibody from Dianova was used. Nuclei were counterstained with Hoechst 33342 (Invitrogen). For cell death analysis the In Situ Cell Death Detection Kit for TUNEL (TdT-mediated dUTP nick end labelling) from Roche was used. Bright-field and fluorescence pictures were taken by using the DMI4000 B microscope (Leica) in combination with a LEICA DFC360 FX or LEICA DFC420C camera.

### Histology Scoring

Pathology scoring was performed on H&E stained small intestinal tissue sections by averaging the total amount of (a) Integrity of the intestinal epithelium: intact and no pathological changes (0), mild, moderate, severe destruction (1,2,3) and (b) Mucosal inflammation: no inflammatory infiltrates (0), rare, moderate or massive invasion of immune cells (1,2,3).

### Organoid culture and treatment

As described by Sato *et al*., crypts were cultured and grown to organoids for at least 5 days [19]. To induce *cFlip* deletion and/or *vFlip* expression, organoids were treated for three days with 50 ng tamoxifen/mL cell culture medium. On the third day, organoids were further treated with TNFα (25 ng/mL; Immunotools). To reveal dying cells, organoids were stained with Propidiumiodide (1:300, BD Biosciences) during the TNFα treatment.

### Gene expression

Total RNA was isolated from the gut tissue by using the Peqgold Total RNA Kit (C-Line) by Peqlab. cDNA was synthesized by using the Script cDNA Synthesis Kit (Jena Bioscience) and analyzed by quantitative real time PCR with SYBRgreen reagent from Roche and QuantiTect primer assays by Qiagen. The results were normalized to the expression levels of the housekeeping gene *hypoxanthine guanine phosphoribosyl transferase* (*Hprt*) or *Glyceraldehyde 3-phosphate dehydrogenase* (*Gapdh)*.

### Immunoblotting

Proteins were isolated from intestinal epithelial cells as previously described [20], separated by using a MiniProtean Precast gel (4–15% polyacrylamide, Bio-Rad) and transferred to a nitrocellulose membrane (Millipore). For protein detection, an anti-GFP and a directly-labeled anti-Actin antibody (both abcam) were used. As a secondary antibody, an HRP-linked anti-rabbit antibody from Cell Signaling was used.

### Statistical analysis

Statistical analysis was performed using the two-tailed student T-test and one way ANOVA. *p<0.05, **p<0.01, ***p<0.001.

## Results

### *vFlip* expression restores the phenotype induced by *cFlip* deletion

**Fig 1:**
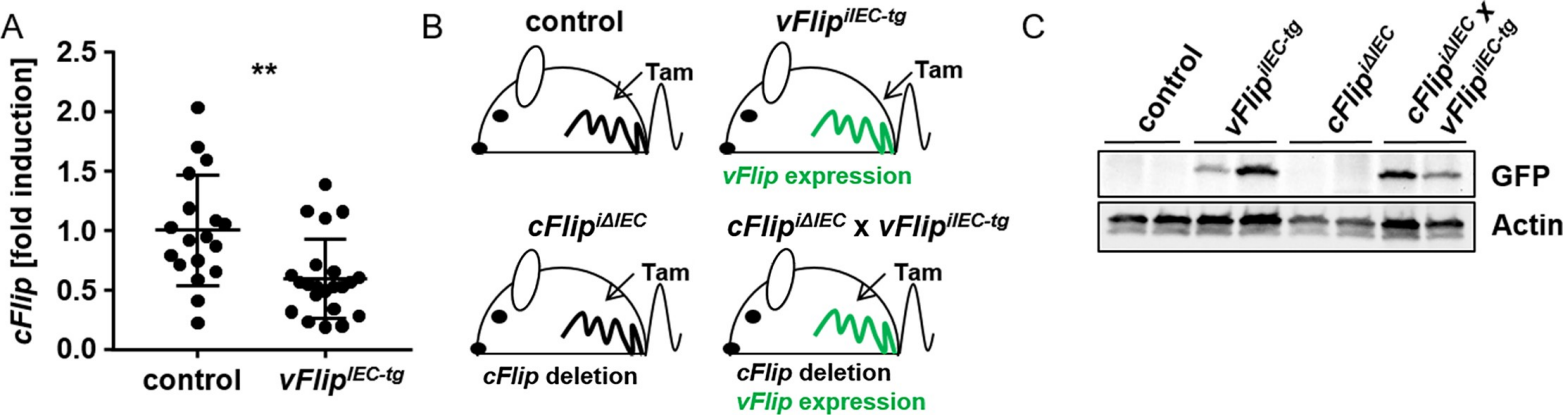
Analysis of vFLIP-regulated *cFlip* expression and generation of an inducible mouse model. (A) Gene expression analysis of *cFlip* in control and *vFlip*^*IEC-tg*^ mice; n≥18. Fold induction values were calculated relative to controls, *Hprt* was used as a housekeeping gene. (B) Generation of mouse models to induce *vFlip* expression (linked to *Gfp* expression) and/ or to delete *cFlip* expression respectively in IECs of mice after at least 2 days of consecutive intraperitoneal tamoxifen injection. (C) Western Blot analysis of GFP in small intestinal lysates of indicated mice after 2 consecutive days of tamoxifen injection. Actin was used as a loading control.

**Fig 2:**
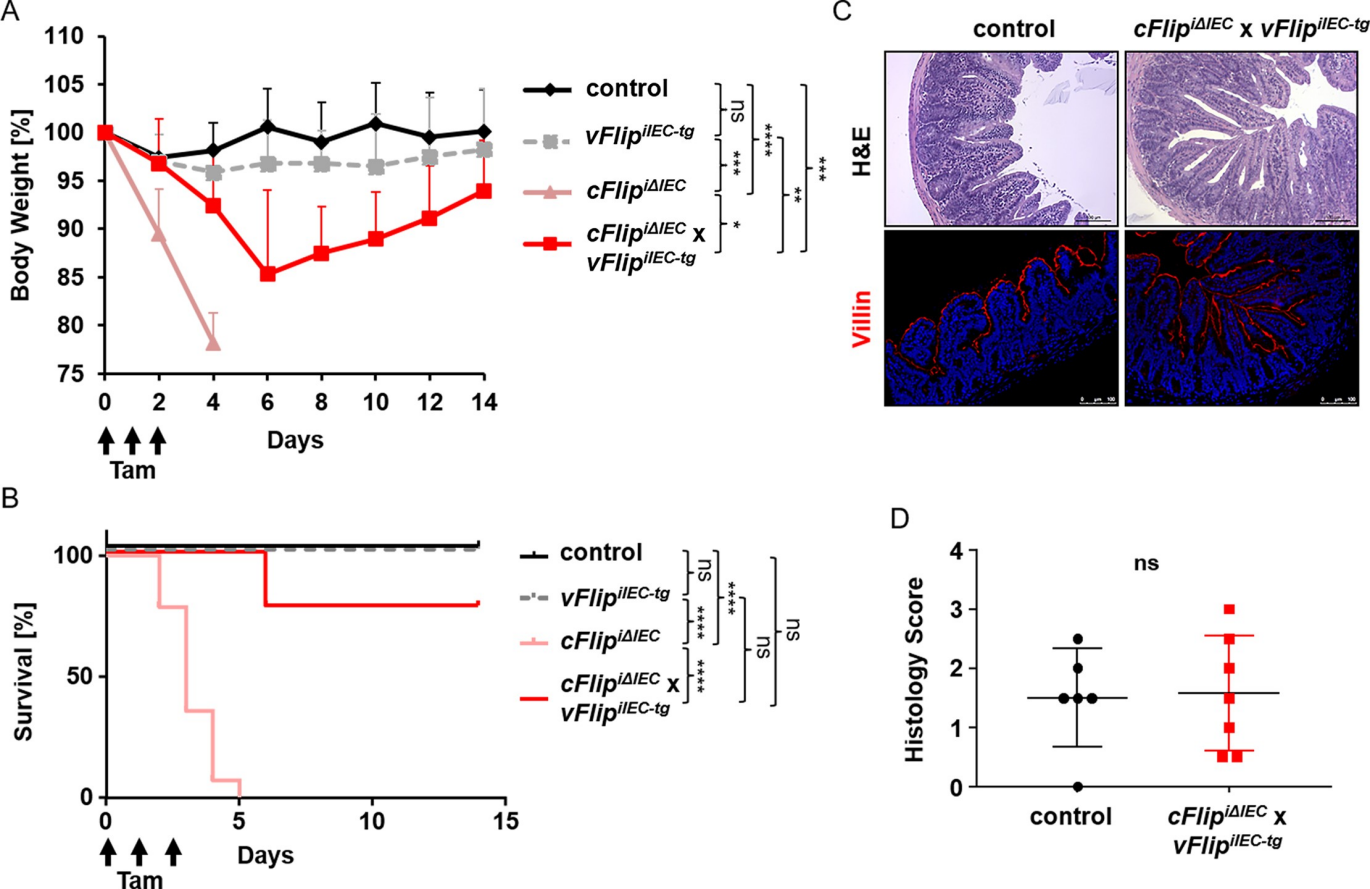
*vFlip* expression largely protects from lethality induced by *cFlip* deletion. (A) Kaplan-Meier Survival and (B) body weight curve of control (n = 10), *vFlip*^*iIEC-tg*^ (n = 8), *cFlip*^*iΔIEC*^ (n = 13) and *cFlip*^*iΔIEC*^ *x vFlip*^*iIEC-tg*^ (n = 9) mice injected with tamoxifen for 3 consecutive days. Mice were euthanized due to increased body weight loss (>20%) over the 14 days period upon tamoxifen treatment. (C) Representative picture of H&E and immunohistochemical staining of Villin (red) on cross sections of surviving control and *cFlip*^*iΔIEC*^ *x vFlip*^*iIEC-tg*^ mice. Nuclei were counterstained with Hoechst 33342. Scale bar = 100μm. (D) Histology score of whole H&E stained cross sections of control (n = 6) and *cFlip*^*iΔIEC*^ *x vFlip*^*iIEC-tg*^ (n = 7) mice.

Previous results generated by our group showed that mice which express HHV8-*vFlip* in intestinal epithelial cells (*vFlip*^*IEC-tg*^ mice) are characterized by spontaneous intestinal inflammation accompanied by Paneth cell loss, in addition to massive epithelial cell death and constitutive activation of the NFκB pathway [16]. Although the *cFlip* gene was described to be a target of the NFκB pathway [21, 22], *vFlip*^*IEC-tg*^ mice showed a significantly decreased *cFlip* gene expression in the small intestine as compared to controls (Fig 1A). Due to these results, we suggested that *cFlip* expression is actively downregulated during viral HHV8 infection. This means that HHV8-vFLIP could adopt cFLIP functions in the host cell to deregulate and adjust the cell death and survival machinery. To analyze if HHV8-vFLIP is able to compensate or regulate the function of cFLIP in the cell, we generated mice with an inducible expression of *vFlip* together with a deletion of *cFlip* in IECs (*cFlip*^*iΔIEC*^ *x vFlip*^*iIEC-tg*^ mice). These animals were compared to control mice and mice which were characterized by IEC-specific inducible *cFlip* deletion (*cFlip*^*iΔIEC*^ mice) or *vFlip* expression (*vFlip*^*iIEC-tg*^ mice) respectively (Fig 1B). Induction of *vFlip* gene expression and therefore a functional inducible model was confirmed by western blot analysis of GFP, which is accompanied by *vFlip* expression [17] (Fig 1C, S1–S3 Figs). After confirming the successful modification of gene expression in IECs, in a next experiment, we monitored body weight and survival of *cFlip*^*iΔIEC*^ x *vFlip*^*iIEC-tg*^ mice, as well as of controls, *cFlip*^*iΔIEC*^ knockout mice and *vFlip*^*iIEC-tg*^ transgenic mice after subsequent tamoxifen injection over time. In accordance to our previous study [6], all control mice survived the indicated time period of treatment whereas all *cFlip*^*iΔIEC*^ mice showed dramatic weight loss and died within 5 days (Fig 2A and 2B). As expected, all of the *vFlip*^*iIEC-tg*^ mice survived, as even mice with a constitutive *vFlip* expression in IECs were viable [16]. Strikingly, *cFlip*^*iΔIEC*^ x *vFlip*^*iIEC-tg*^ mice were characterized by a significantly higher survival rate as compared to *cFlip*^*iΔIEC*^ mice and most of the animals could recover from severe body weight loss around day 8 (Fig 2A and 2B). Interestingly, histological analysis of these surviving *cFlip*^*iΔIEC*^ x *vFlip*^*iIEC-tg*^ mice showed no signs of increased cell death and depicted a regular crypt-villus architecture (Fig 2C and 2D). These findings were further underlined by immunohistochemical staining of Villin, revealing an intact epithelial barrier comparable to controls (Fig 2C). In summary, these experiments show that *vFlip* expression in IECs largely protects from lethality and body weight loss induced by excessive cell death, suggesting that vFLIP might counteract apoptosis induced by the host as a defense reaction to prevent viral propagation.

### vFLIP protects from increased cell death and loss of intestinal barrier integrity in the absence of cFLIP

**Fig 3:**
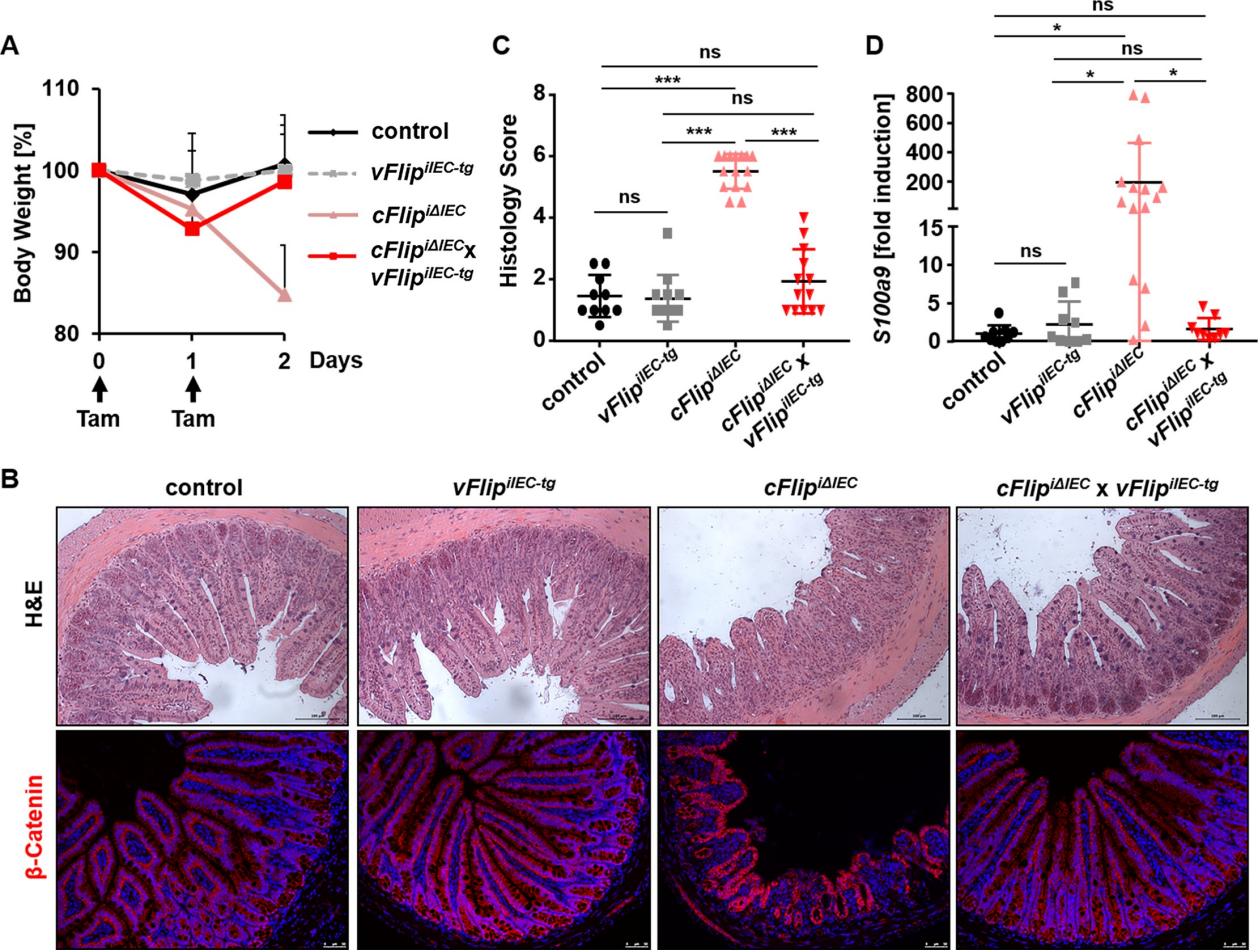
vFLIP maintains intestinal barrier integrity in a short term model *in vivo*. (A) Body weight curve of control (n = 11), *vFlip*^*iIEC-tg*^ (n = 12), *cFlip*^*iΔIEC*^ (n = 16) and *cFlip*^*iΔIEC*^ *x vFlip*^*iIEC-tg*^ (n = 14) mice injected with tamoxifen for 2 consecutive days. Experiment was ended one day later. (B) Representative pictures of H&E and β-Catenin (red) staining of indicated mice. Nuclei were counterstained with Hoechst 33342. Scale bar upper panel 100μm, lower panel 50μm. (C) Histology score of whole H&E stained cross sections of control (n = 10), *vFlip*^*iIEC-tg*^ (n = 12), *cFlip*^*iΔIEC*^ (n = 15) and *cFlip*^*iΔIEC*^ *x vFlip*^*iIEC-tg*^ (n = 13) after two consecutive days of tamoxifen treatment. (D) Gene expression analysis of *S100a9* in small intestinal lysates of of control (n = 9), *vFlip*^*iIEC-tg*^ (n = 9), *cFlip*^*iΔIEC*^ (n = 15) and *cFlip*^*iΔIEC*^ *x vFlip*^*iIEC-tg*^ (n = 9) mice after two days of consecutive tamoxifen injection. Fold induction values are calculated relative to controls, *Gapdh* was used as housekeeping gene.

It is apparent that cFLIP is an essential regulator in early life phases, as general genetic deletion of *cFlip* leads to embryonic lethality around day e10.5 [23]. Moreover, the observation that IEC-specific *cFlip*-deficient mice are not viable [6], suggests an important role of *cFlip* during gut development. To further analyze the ability of vFLIP to compensate for cFLIP with regard to cell death inhibition, we injected control, *vFlip*^*iIEC-tg*^, *cFlip*^*iΔIEC*^ and *cFlip*^*iΔIEC*^ *x vFlip*^*iIEC-tg*^ mice on two subsequent days (day 0, 1) with tamoxifen and sacrificed them one day later (day 2). Surprisingly, *cFlip*^*iΔIEC*^ *x vFlip*^*iIEC-tg*^ mice showed less weight loss when compared to *cFlip*^*iΔIEC*^ mice (Fig 3A) at day 2 in this short term model. Further histological analyses via H&E staining and histology score revealed major differences between *cFlip*^*iΔIEC*^ *x vFlip*^*iIEC-tg*^ and *cFlip*^*iΔIEC*^ mice (Fig 3B and 3C). Whereas the intestinal barrier and crypt-villus structure were severely destroyed in *cFlip*^*iΔIEC*^ mice, *cFlip*^*iΔIEC*^ *x vFlip*^*iIEC-tg*^ mice showed a regular crypt-villus architecture comparable to controls (Fig 3B and 3C). Immunohistochemical staining of β-Catenin moreover confirmed that the intestinal epithelium in *cFlip*^*iΔIEC*^ *x vFlip*^*iIEC-tg*^ mice was intact and comparable to controls, whereas *cFlip*^*iΔIEC*^ mice were characterized by a disrupted epithelial barrier (Fig 3B). In accordance and in strong contrast to *cFlip*^*iΔIEC*^ mice, *cFlip*^*iΔIEC*^ *x vFlip*^*iIEC-tg*^ mice showed significantly lower gene expression levels of the proinflammatory marker *S100a9*, comparable to controls (Fig 3D). Altogether these results show that expression of *vFlip* counteracts disruption of the epithelial barrier and additionally reduces the inflammatory response provoked by *cFlip* deletion in IECs.

### *vFlip* expression protects from Caspase-8 mediated apoptosis

**Fig 4:**
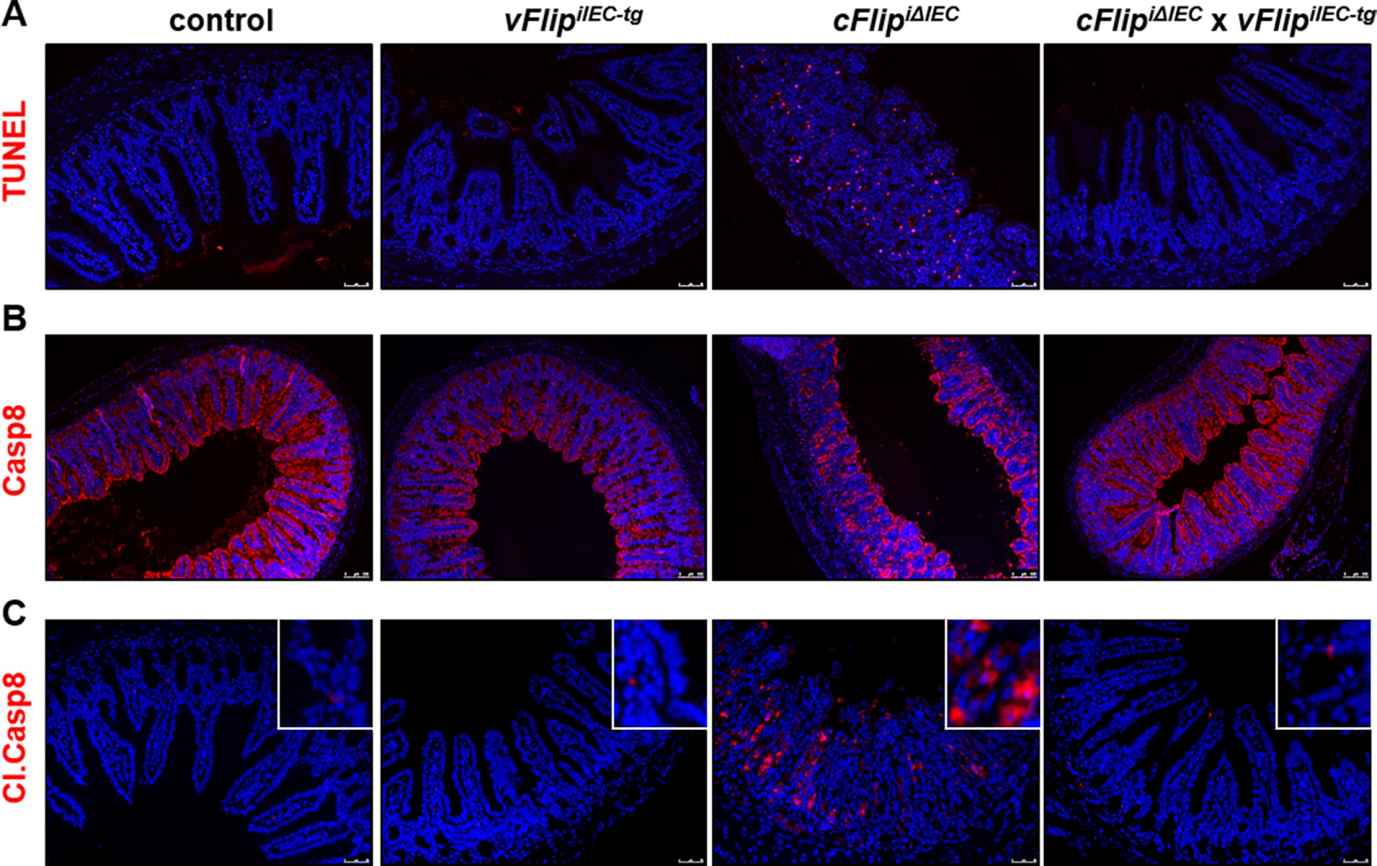
Epithelial cell death induced by cFlip deletion is counteracted by vFLIP *in vivo*. Representative pictures of (A) TUNEL, (B) Caspase-8 and (C) Cl. Caspase-8 stainings (all red) on cross-sections of indicated mice. Nuclei were counterstained with Hoechst 33342. Scale bar upper and lower panel 50μm, middle panel 100μm.

As described earlier, *cFlip*^*iΔIEC*^ mice are characterized by loss of barrier integrity due to massive epithelial cell death [6]. Cell death induced by *cFlip-*deficiency was identified as apoptosis, which was mediated by amplified unregulated Caspase-8 activity. To analyze if vFLIP protects from uncontrolled IEC death in this model, we performed TUNEL staining on small intestinal cross-sections of mice from all four groups. Staining revealed that *vFlip* expression on a *cFlip*-deficient background protects from excessive epithelial cell death in the gut, suggesting that vFLIP counteracts apoptosis induced by *cFlip*-deficiency (Fig 4A). These results were underlined by an additional immunohistochemical staining showing that signals of cleaved Caspase-8 were clearly lower in IECs of *cFlip*^*iΔIEC*^ *x vFlip*^*iIEC-tg*^ mice as compared to *cFlip*^*iΔIEC*^ mice (Fig 4C). Of note protein levels of uncleaved Caspase-8 were comparable among all four groups (Fig 4B).

**Fig 5:**
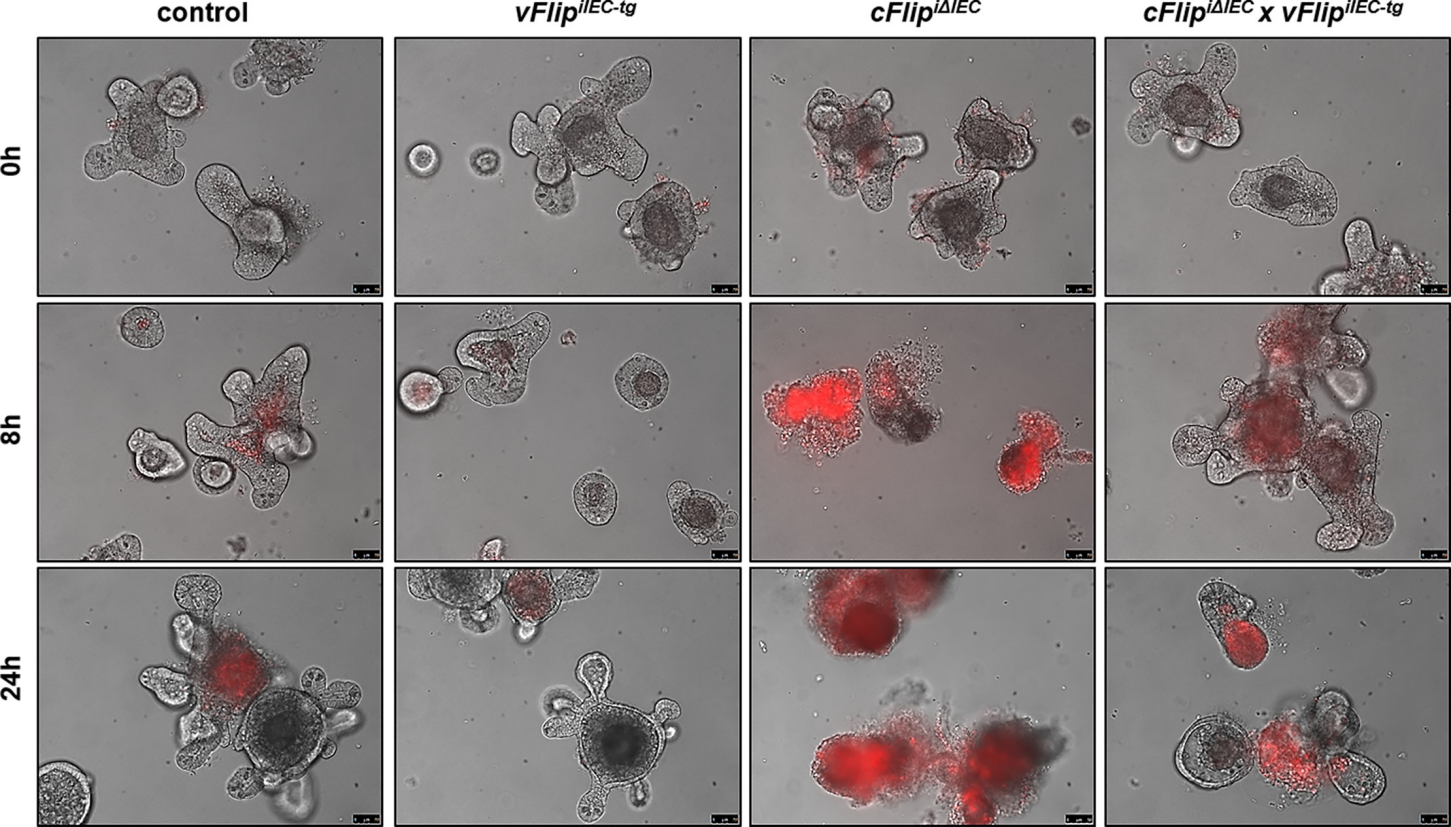
*vFlip* expression protects from TNFα induced cell death in *cFlip*-deficient organoids. Representative microscopic pictures (bright field) of small intestinal organoids derived from indicated mice, treated with tamoxifen (50ng/ml) for 4d and afterwards stimulated with TNFα (25ng/ml) for indicated time. Dead cells were stained with propidium iodide (red), Scale bar = 75μm.

To analyze the effect of viral FLIP on cFLIP-regulated cell death specifically in IECs independent of other cell types, we generated epithelial organoids derived from the small intestine of the four different mouse groups. Without additional stimulation, all organoids were viable and characterized by an intact epithelial architecture (Fig 5 upper panel). Interestingly, *cFlip*-deficient organoids were more susceptible towards TNFα-induced cell death than organoids derived from *cFlip*^*iΔIEC*^ *x vFlip*^*iIEC-tg*^ mice. Only 8h after TNFα treatment, organoids derived from *cFlip*^*iΔIEC*^ mice were characterized by increased cell death. This was indicated by propidium iodide staining, as well as by the loss of 3D structure and epithelial integrity (Fig 5 middle panel). 24h after TNFα stimulation, all *cFlip*-deficient organoids were dead, whereas organoids from all other mouse groups survived (Fig 5 lower panel). In summary these organoid results *in vitro* underline the results obtained in the *in vivo* experiments. Our data shows that vFLIP mediates survival functions *in vivo* and *in vitro* to counteract excessive apoptotic cell death induced by uncontrolled Caspase-8 activation mediated by loss of *cFlip*.

## Discussion

**Fig 6:**
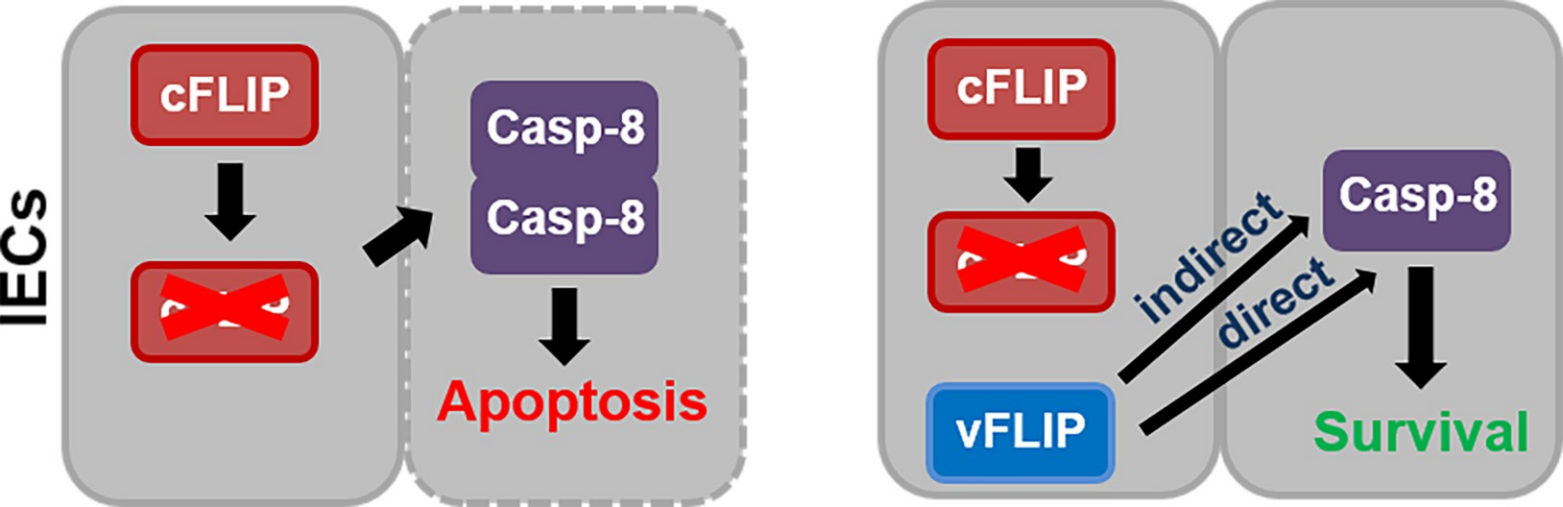
Potential influence of vFLIP in a short term apoptosis model. *cFlip*-deficiency in IECs mediates uncontrolled Caspase-8 activation culminating into increased apoptosis. Additional *vFlip* expression during viral infection might either directly or indirectly via NFκB compensate for loss of cFLIP, finally blocking apoptotic cell death to circumvent host defense reactions.

In the present study, we show for the first time that expression of a viral FLIP protein counteracts apoptotic death in the gut, which is induced by loss of the cell death regulator cFLIP. Since HHV8-vFLIP and cFLIP share structural homologies and highly similar amino acid sequences [24], one might speculate that vFLIP as well as cFLIP_short_ bind to Caspase-8 and directly regulate its activity by blocking its cleavage. The ability of vFLIP to block the Caspase-8 activation was shown *in vitro* by Bélanger *et al*. [14]. However, HHV8-vFLIP is also a known potent activator of NFκB signaling [25, 26]. vFLIP directly binds to the IKKγ/NEMO-complex [24, 27], inducing the phosphorylation and proteasomal degradation of the IκB inhibitor, further enabling NFκB proteins to translocate to the nucleus and activate target gene expression. Interestingly, classical NFκB signaling is described to induce gene expression of pro-survival genes, which counteract cell death [28]. Surprisingly, although *cFlip* was described as a gene, which is induced in response to NFκB activation [21, 22], our data shows that constitutive expression of *vFlip* in intestinal epithelial cells *in vivo* significantly reduced gene expression of *cFlip* in the gut. Therefore one might conclude that *cFlip* is not only regulated indirectly by NFκB, but also by other mechanisms that are cell-intrinsically mediated by *vFlip*. In accordance, in our previous study, we could show that vFLIP is not only located in the cytoplasm, but also in the nucleus of transfected mammalian cells, suggesting that vFLIP might directly alter gene transcription in target cells [29]. Moreover one might suggest that vFLIP, which is expressed in intestinal epithelial cells, influences *cFlip* expression in the gut also indirectly via microenvironmental factors, e.g. cytokines. These cytokines could further activate other signaling pathways beside NFκB, that regulate *cFlip* expression in cells adjacent to epithelial cells. Beside constitutive activation of NFκB, constitutive expression of *vFlip* in IECs moreover induced the spontaneous development of intestinal inflammation accompanied by Paneth cell loss and increased epithelial cell death [16]. However, the phenotype of *vFlip*^*IEC-tg*^ mice was not completely identical to mice with a constitutive activation of NFκB in IECs [30, 31], which were not characterized by increased cell death. One might reason, that vFLIP not only indirectly via NFκB pro-survival signals, but also directly by interaction, regulates Caspase-8 activation and the host cell death machinery (Fig 6). These findings lead to the hypothesis that viruses via *vFlip* expression might actively downregulate *cFlip*, potentially independently of NFκB, for improved regulation of the host cell death machinery during infection. This is in line with previous publications showing HSV1-induced reduction of cFLIP in several cell types, e.g. dendritic cells and epithelial cells [32, 33]. However, expression of HHV8-*vFlip* in PEL cells increases protein levels of the long cFLIP isoform, suggesting cell-type specific mechanisms of cFLIP regulation [34]. Interestingly our present results reveal that short-term expression of *vFlip* in IECs counteracts cell death induced by *cFlip*-deficiency, potentially to gain more time for efficient replication during viral infection and to avoid host defense reactions. In contrast, constitutive *vFlip* expression not only blocks apoptosis but additionally induces a rather necrotic type of cell death [16], which might facilitate viral propagation after successful replication. For the first time ever, this study compares the ability of viral and cellular FLIP to regulate the cell death machinery in the gut *in vivo* in a short term apoptosis model and *in vitro* in the organoid model. Our data suggests that viral FLIP is able to deregulate the host cell death machinery in epithelial cells by changing the gene expression of host cell death regulators and by blocking activation of the apoptosis cascade, which might play an important role for the pathogenesis of viral infections of mucosal surfaces.

## Supporting information

S1 Fig Original Blot data. (PDF)

S2 Fig Original Blot data_Actin. (TIF)

S3 Fig Original Blot data_ Gfp. (TIF)
