# Supplementary figures and images for: Viral FLIP blocks Caspase-8 driven apoptosis in the gut in vivo
Source: PLoS One. 2020 Jan 30;15(1):e0228441. doi: 10.1371/journal.pone.0228441 (PMC6992192; doi:10.1371/journal.pone.0228441)

Original Blots, Fig. 1C

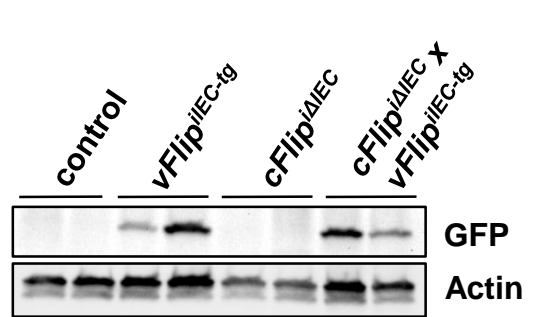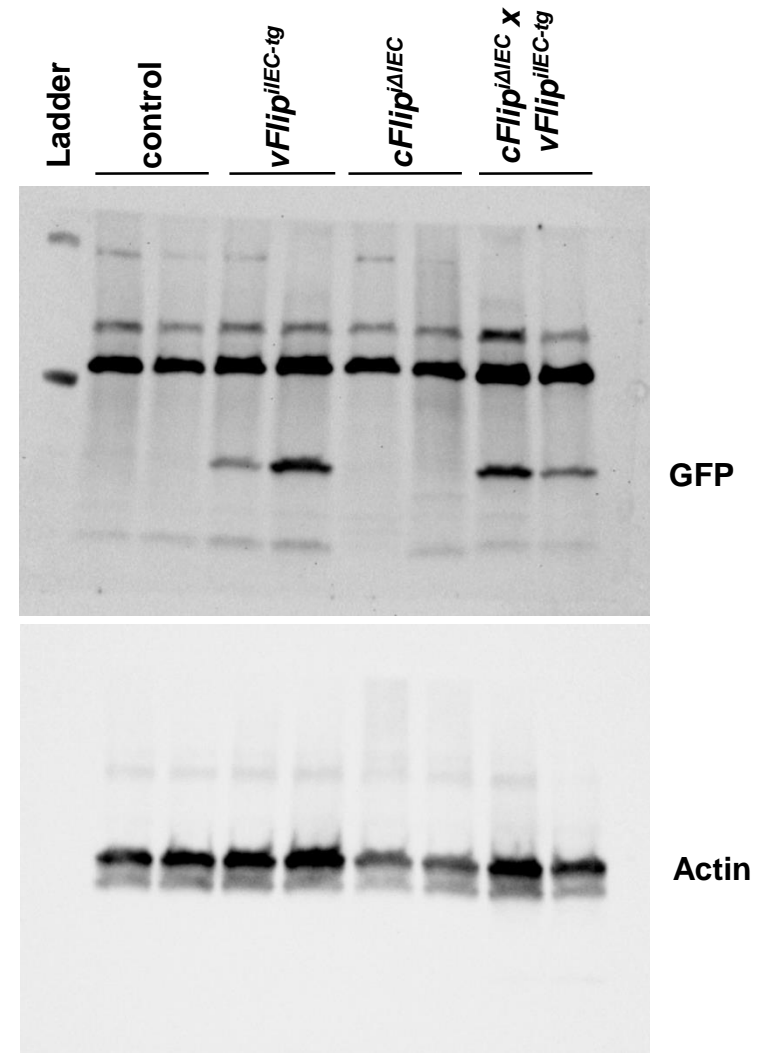

Supplement: S1 Fig — (PDF) [file pone.0228441.s001.pdf]

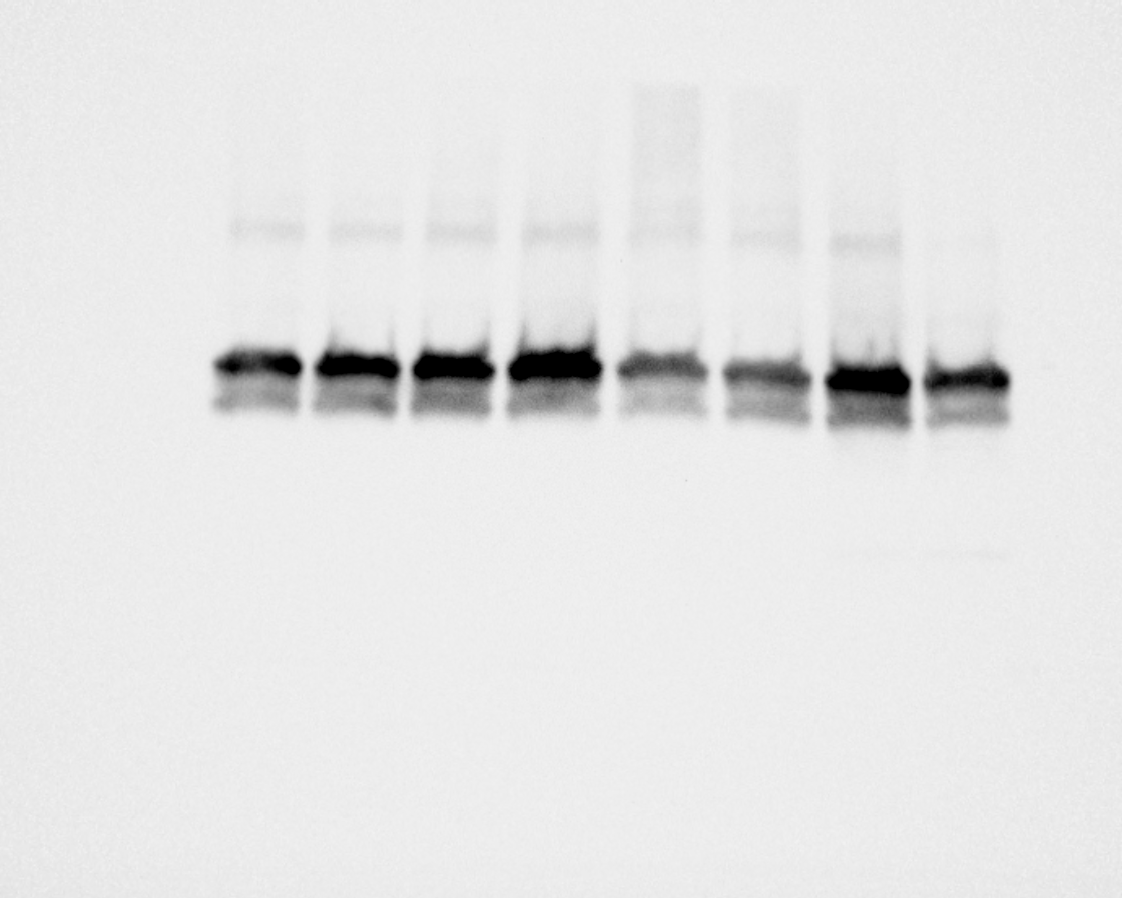

Supplement: S2 Fig — (TIF) [file pone.0228441.s002.tif]

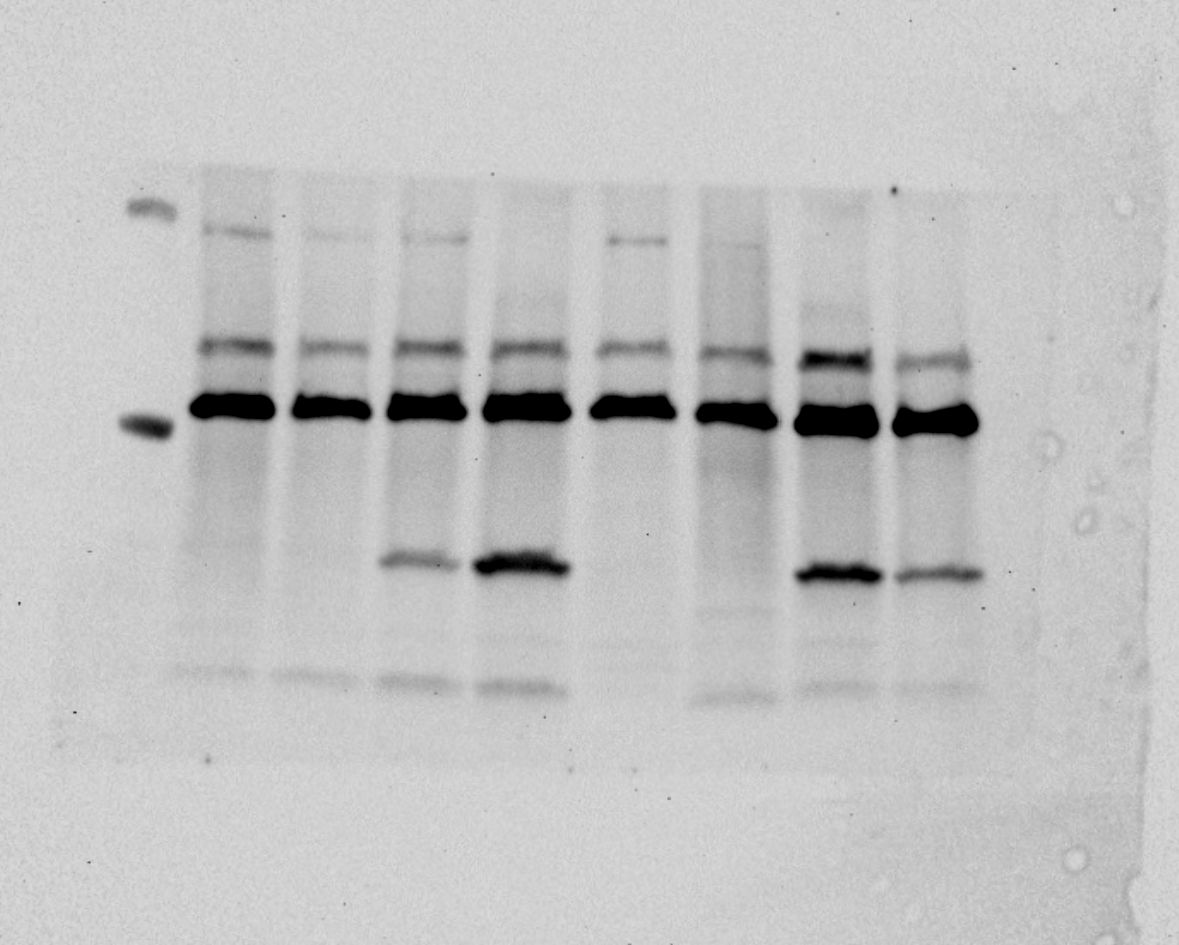

Supplement: S3 Fig — (TIF) [file pone.0228441.s003.tif]
